# A Novel Rhabdovirus Associated with the Idaho Population of Potato Cyst Nematode *Globodera pallida*

**DOI:** 10.3390/v14122718

**Published:** 2022-12-05

**Authors:** Joanna Kud, Jennifer Dahan, Gardenia E. Orellana, Louise-Marie Dandurand, Alexander V. Karasev

**Affiliations:** 1 Department of Entomology, Plant Pathology and Nematology, University of Idaho, Moscow, ID 83844-2329, USA; 2 Department of Entomology and Plant Pathology, University of Arkansas, Fayetteville, AR 72701-4006, USA; 3 Bioinformatics and Computational Biology Program, 875 Perimeter Drive, MS 1103, University of Idaho, Moscow, ID 83844-1103, USA

**Keywords:** potato cyst nematode, *Globodera pallida*, *Rhabdoviridae*, negative-sense single-stranded RNA virus, biocontrol

## Abstract

*Globodera pallida*, a potato cyst nematode (PCN), is a quarantine endoparasitic pest of potato (*Solanum tuberosum*) in the US due to its effects on yield and quality of potato tubers. A new rhabdovirus, named potato cyst nematode rhabdovirus (PcRV), was revealed and characterized in the *G. pallida* populations collected in Idaho through use of high-throughput sequencing (HTS) and RT-PCR and found to be most closely related to soybean cyst nematode rhabdovirus (ScRV). PcRV has a 13,604 bp long, single-stranded RNA genome encoding five open reading frames, including four rhabdovirus-specific genes, N, P, G, and L, and one unknown gene. PcRV was found present in eggs, invasive second-stage juveniles, and parasitic females of *G. pallida*, implying a vertical transmission mode. RT-PCR and partial sequencing of PcRV in laboratory-reared *G. pallida* populations maintained over five years suggested that the virus is highly persistent and genetically stable. Two other *Globodera* spp. reproducing on potato and reported in the US, *G. rostochiensis* and *G. ellingtonae*, tested negative for PcRV presence. To the best of our knowledge, PcRV is the first virus experimentally found infecting *G. pallida*. Based on their similar genome organizations, the phylogeny of their RNA-dependent RNA polymerase domains (L gene), and relatively high identity levels in their protein products, PcRV and ScRV are proposed to form a new genus, provisionally named “Gammanemrhavirus”, within the family *Rhabdoviridae*.

## 1. Introduction

*Globodera pallida*, a potato cyst nematode (PCN), is a globally regulated plant parasitic nematode [1] that may reduce tuber yields up to 80% in infested potato (*Solanum tuberosum*) fields if left unmanaged [2,3]. *G. pallida* was first reported in the United States in Idaho in 2006 [4], and, due to its devastating economic impact on the potato industry, was immediately placed under strict quarantine and eradication programs [5]. Due to the high toxicity of chemical nematicides, such as methyl bromide, it is desirable to develop new, safer alternative strategies to eradicate or reduce nematode populations in the field. Restrictions on use of chemical pesticides have put studies on alternative methods of nematode control in the spotlight. Among safer alternatives are biological control agents (BCAs). Many examples show that viruses are viable as BCAs for limiting the impact phytopathogens, such as fungi, bacteria, insects, and even other viruses, have on crops [6].

Viruses are obligate intracellular parasites found in all organisms that sometimes cause diseases in their hosts, and, if found in nematodes, could be explored as a potential tool for their control. One putative, positive-strand RNA virus was recently discovered among transcriptomics outputs from *G. pallida*, potato cyst nematode picorna-like virus (PcPLV) [7]. This transcriptomic library was generated from *G. pallida* and *G. rostochiensis* populations collected in Scotland [8,9], and the virus presence could not be confirmed experimentally in the US populations of these nematodes due to the quarantine status of *G. pallida* and *G. rostochiensis* in the US [7]. Hence, experimental evidence of the PcPLV virus presence in laboratory or field populations of *G. pallida* and *G*. *rostochiensis* is still lacking. Earlier this year, we published a transcriptomics study of *G. pallida* collected in the State of Idaho (USA) [10] and, in search of potential tools to manage *G. pallida*, decided to subject this extensive dataset to a bioinformatics analysis focused on discovery of viruses possibly present in the Idaho populations.

Here, we report on a novel rhabdovirus genome assembled from the transcriptomics reads available from the database [10]. The presence of this virus, named potato cyst nematode rhabdovirus (PcRV), was validated and confirmed for the different life stages of the *G. pallida* populations maintained in the quarantine greenhouse in Idaho (USA), and the virus whole genome was re-sequenced using Sanger methodology. Another isolate of PcRV was identified among the publicly available transcriptomics outputs generated from the Scottish populations of *G. pallida*, with the genome sequence almost identical to the Idaho isolate of PcRV. Based on the phylogenetic position of PcRV, we propose to create a new genus with a tentative name Gammanemrhavirus, comprising two virus species, representing PcRV and ScRV, respectively.

## 2. Materials and Methods

### 2.1. Globodera Populations

Three *Globodera* species were used in this study: *G. pallida*, *G. ellingtonae*, and *G. rostochiensis*. *G. pallida* was originally collected in 2006 from an infested potato field in Shelley, ID [4]. *G. ellingtonae* cysts were collected in 2008 from an infested field in Powell Butte, OR and obtained from Dr. Inga Zasada (USDA, ARS, Corvallis, OR, USA) [11]. *G. rostochiensis* cysts were obtained from Dr. Xiaohong Wang (USDA-ARS, Ithaca, NY, USA). For rearing, nematodes were inoculated on ‘Russet Burbank’, a susceptible potato cultivar in a greenhouse under standard conditions (10 ± 2 °C night-time, 18 ± 2 °C day-time, 16:8 h light:dark photoperiod). At 16 weeks post inoculation, the elutriator method [12] was used to extract cysts from the soil. Dried and clean cysts were incubated at 4 °C for a minimum of 16 weeks prior to experimental use.

### 2.2. Obtaining Globodera Juveniles and Females

The cysts were placed in meshed polyvinyl chloride (PVC) tubes nested in 6-well plates, surface sterilized with 0.3% hypochlorous bleach for 5 min followed by five rinses in sterile distilled water [13], and then hydrated in deionized (DI) water for 2 days. After 2 days, the water was replaced with root diffusate collected from ‘Russet Burbank’ plants via a soil percolation method [14] with addition of the antibiotic, gentamicin sulfate, at final concentration of 750 µg/mL (Sigma Aldrich, St. Louis, MO, USA). The plates were incubated at 19 °C for the next 14 days and the hatched second-stage juveniles (J2s) were used for the study. Invasive J2s were collected and washed three times with sterile DI water by centrifugation at 150× *g* for 5 min. Collected J2s were counted under a stereomicroscope (Leica Microsystems CMS GmbH, Wetzlar, Germany) and either used immediately for plant inoculation or frozen in liquid nitrogen and stored at −80 °C for subsequent nucleic acid extraction. ‘Desiree’ plants, also PCN-susceptible, were propagated and transferred under greenhouse conditions as described previously [15]. J2s were surface sterilized in 100 µg/mL of ampicillin, 100 µg/mL of streptomycin, and 0.125% wt/vol benzethoniumchloride [16], resuspended in 0.1% agarose (Agarose I; VWR Life Science, Radnor, PA, USA), and inoculated onto potato at a rate of 1000 J2s per each plant. *G. pallida* females were pooled from roots of ten infected plants 21 days after inoculation according to methods described by Cotton et al. (2014) [8]. Once collection was completed, nematodes were washed twice with sterile DI water and counted under a stereomicroscope; excess supernatant was removed, and the sample was then frozen in liquid nitrogen and stored at −80 °C for subsequent nucleic acid extraction.

### 2.3. Nucleic Acid Extraction, RT-PCR Testing, and Sanger Sequencing

Total RNA was extracted from either 10 cysts (eggs), 1000 J2s, or 500 females using RNeasy Mini Kit Plus (Qiagen, Hildren, Germany) with minor modifications as described by Kud et al. [10]. Two hundred and fifty ng of total RNA was converted into cDNA using Anchored Oligo(dT)_20_ (Invitrogen, Carlsbad, CA, USA) as primers and SuperScript III reverse transcriptase (Invitrogen) according to the manufacturer’s instruction. All PCR reactions were accomplished by *Taq* DNA polymerase (New England BioLabs, Ipswich, MA, USA) in a 20 µL reaction mixture that contained 2 µL 10x buffer, 2.5 mM dNTP, 5 µM of each forward primer and reverse primer, 0.5 unit of *Taq* DNA polymerase, and 1 µL cDNA template. The PCR profile consisted of denaturing at 95 °C for 30 s and 35 cycles of 95 °C for 30 s, 60 °C for 30 s, and 68 °C for 1 min, followed by a final extension for 5 min at 68 °C. Sanger sequencing was performed on a series of overlapping RT-PCR fragments [17] amplified on total RNA extracted from hatched *G. pallida* J2s as described above. Primers used to amplify these DNA fragments are listed in Supplementary Table S1. PCR fragments were treated with ExoSAP-IT (Affymetrix, Cleveland, OH, USA) and submitted for sequencing to Elim Biopharmaceuticals, Inc. (Hayward, CA, USA). The 5′ terminus was amplified using a 5′/3′ RACE kit (Roche, Indianapolis, IN, USA) according to the manufacturer’s instructions using primers SP1, SP2, and SP3 (Supplementary Table S1); the amplified PCR product was cloned into *E. coli* using a pGEM-T Easy vector system (Promega, Madison, WI, USA) and sequenced using M13 primers. The 3′ terminus was amplified using SP5 primer (Supplementary Table S1), then cloned and sequenced with M13 primers. The obtained contigs were assembled using Geneious Prime v2021.1.1 (Biomatters, Auckland, New Zealand).

### 2.4. High-Throughput Sequencing (HTS) and Sequence Analysis

Publicly available transcriptome data of *G. pallida* and *G. ellingtonae* J2s (PRJNA788476 and ERR202422) were retrieved from the NCBI SRA database. These RNAseq data were obtained from poly (A) enriched libraries using paired-end (2 × 150 bp or 2 × 75 bp, respectively) sequencing on the HiSeq2500 (Illumina, San Diego, CA, USA) as described previously [10]. Paired-end reads were assembled using rnaSPAdes v3.14.0 [18] and resulting contigs of size >1 kb were then submitted to BLASTn and BLASTx searches against the GenBank nucleotide sequence databases and against NCBI protein database restricted to virus-related sequences (taxid: 10239), respectively, with a cut-off e-value of 0.0001. A search for conserved protein domains was conducted using the Conserved Domain database (CDD) available at the NCBI [19,20].

### 2.5. Sequence and Phylogenetic Analysis

The phylogenetic tree for the L protein was generated based on the set of sequences provided in the International Committee on Taxonomy of Viruses (ICTV) report on the family *Rhabdoviridae* [21], with the addition of the PcRV (this work) and ScRV (HM849039) [22] L-protein sequences. Tree was generated as described in the ICTV report using the original alignment made available to the public [21]. Maximum clade credibility (MCC) tree was inferred from MUSCLE alignments of full-length rhabdovirus L sequences. Ambiguously aligned amino acid residues in each alignment were pruned using Gblocks [23]. MCC trees were inferred in BEAST.v1.10.4 by using the Whelan and Goldman (WAG) model of amino acid substitutions, the gamma + invariant sites model of site heterogeneity, and a strict molecular clock (coalescent: constant size) with a random starting tree to perform 10 million MCMC runs. The analysis was sampled at every 10,000 states. Tree Annotator v1.10.4 was used to output the results of the MCC tree model and calculate posterior probabilities with a burn-in of 1 million states. FigTree was then used to plot the MCC phylogenetic tree.

## 3. Results

### 3.1. Identification of the Virus Genome in the G. Pallida Transcriptome Data and Confirmation of Viral Presence in the Nematodes

**Figure 1:**
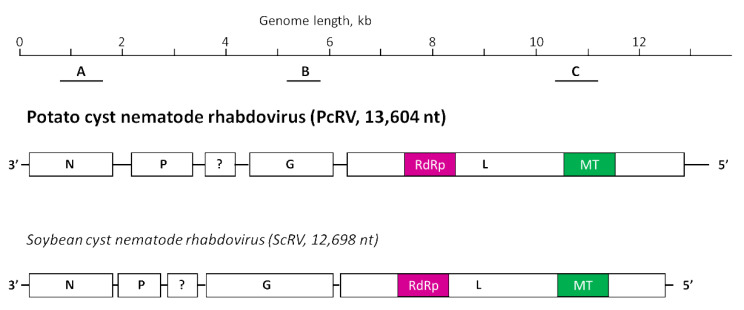
Schematic representation of the potato cyst nematode rhabdovirus (PcRV, GenBank accession number OP903920) genome, presented in a negative-sense virion form, in comparison to the genome organization of soybean cyst nematode rhabdovirus (ScRV, HM849039). Four rhabdovirus-specific genes, N, P, G, and L, are designated; a question mark is placed on an unknown gene. Homologous protein domains encoded in L proteins of both virus genomes, RdRP (RNA-dependent polymerase) and methyltransferase (MT), are also marked with like colors. The black bars below the PcRV genome represent the location of the PCR fragments obtained using the primer pairs for testing by RT-PCR (A, PcRV1_F/R; B, PcRV2_F/R; C, PcRV3_F/R); for primer details, see Supplementary Table S1.

When the Idaho transcriptomics dataset PRJNA788476 was analyzed, a large 13,566 nt contig was assembled from the RNAseq reads, which did not produce any hits when subjected to a BLASTn search through the GenBank database. This 13,566 nt contig was assembled as a negative-strand genome and encoded five open reading frames (ORFs) in its reverse complement strand, encoding proteins ranging between 180 (ORF 3) and 2180 (ORF 5) amino acids (aa) in length. In the BLASTx searches through the GenBank databases, significant similarity was noted between the largest ORF 5-encoded protein and L-proteins (RdRPs) of rhabdoviruses, in particular between the L-protein of the soybean cyst nematode rhabdovirus (ScRV; AEF56733.1) with 34.6% identity and 40% coverage. An additional search through the CDD conserved domain database [20] identified two conserved domains, an RdRP domain characteristic of mononegavirales and an mRNA (guanine-7-) methyltransferase (G-7-MTase) domain also characteristic of viruses with negative RNA genome. The overall organization of ORFs in this 13,566 nt contig resembled the putative rhabdovirus-encoded genes for a nucleoprotein (N), a phosphoprotein (P), an unknown (?) gene, a glycoprotein (G), and a putative RdRP or a large nonstructural protein (L) [24]. Presumably, this large contig represented a complete or nearly complete genome of a new rhabdovirus infecting PCN, where the transcriptomics outputs came from. We named this hypothetic virus an Idaho isolate of the potato cyst nematode rhabdovirus (PcRV-Id); its genome is presented in Figure 1 in a side-by-side comparison to the most closely related virus, ScRV.

In pair-wise comparisons between individual PcRV and ScRV proteins, the amino acid sequence identity ranged between 21.2% (ORF1, N-gene) and 34.7% (ORF 5, L-gene) (Table 1). These relatively low albeit detectable levels of sequence conservation in four of the five proteins encoded by PcRV and ScRV suggest possible conservation of the functions in these four proteins, N, P, G, and L, for both virus-nematode systems. ORF 3-encoded protein product did not display any significant similarity between PcRV and ScRV, possibly reflecting differences in functional specialization of the two proteins.

To check if PcRV sequences might have been present in Scottish transcriptomics dataset ERR202422 [8], it was subjected to bioinformatics analysis, as outlined in Materials and Methods. To our surprise, 2912 PcRV-specific reads were extracted that covered a nearly complete virus genome, 13,506 nt; this was deposited in the GenBank under accession number BK062887. The genome of this Scottish isolate of PcRV (PcRV-Sc) shared 99.64% nucleotide sequence identity with the Idaho isolate of PcRV (PcRV-Id) except just four SNPs, an 18 nt deletion in the 5′-UTR, and a 24 nt insertion in an untranslated region between the P-gene and the unknown gene (see Figure 1). The coding capacity of the PcRV-Sc genome was identical to PcRV-Id. Interestingly, previous analyses of the same dataset did not reveal PcRV genome or PcRV-specific reads [7].

**Figure 2:**
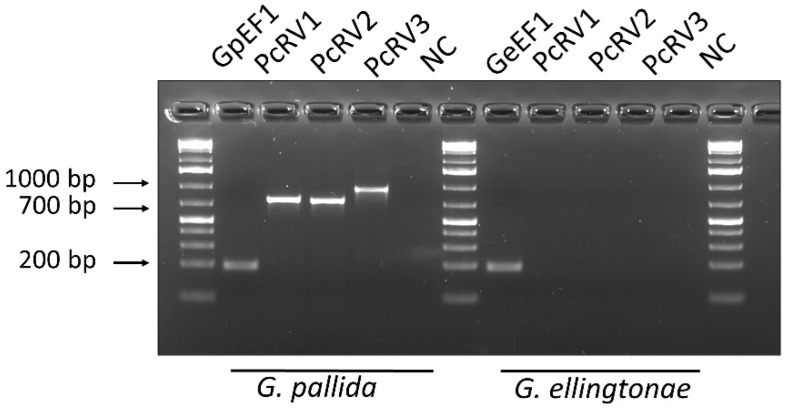
Detection of potato cyst nematode rhabdovirus (PcRV) by endpoint RT-PCR from *Globodera pallida* and *G. ellingtonae* J2s cDNA. Three primer pairs, PcRV1, PcRV2, and PcRV3, described in Materials and Methods, targeted three different PcRV genome regions. The expected sizes of the amplification products were 761 bp, 746 bp, and 947 bp, respectively. Nematode *EF1* gene-specific primer pairs (*GpEF1* or *GeEF1*; 193 bp) served as a positive control for each RT-PCR test. NC—negative non-template H_2_O control. The sequences of primers are listed in Supplementary Table S1.

To confirm experimentally the presence of this putative new virus in samples used for HTS in Idaho, conventional RT-PCR was conducted on subsamples of *G. pallida* and *G. ellingtonae* RNA previously obtained for the original transcriptomic analysis [10]. RT-PCR amplification from *G. pallida* RNA samples using three sets of primers specific for PcRV (PcRV1-3; Figure 1; Supplementary Table S1) resulted in PCR products with expected sizes of 761, 746, and 947 nt, respectively. Direct Sanger sequencing of these PCR products resulted in a complete match to the PcRV sequence derived from the HTS. Although few *G. ellingtonae* HTS reads from the PRJNA788476 transcriptomic dataset aligned with the PcRV genome, *G. ellingtonae* cDNA tested negative in all three PcRV-specific amplifications (Figure 2). To validate the HTS-derived PcRV genome assembly, its genome was Sanger-sequenced. A total of 19 primer pairs were designed to cover the PcRV genome in a series of 19 overlapping PCR-fragments (PcRV_SS1-19; Supplementary Table S1). All the amplification products were then sequenced in both directions and assembled. The 5′ and 3′ termini of the PcRV genome were acquired using the 5′/3′ RACE methodology. The 3′ terminus was Sanger-sequenced directly from the 3′ RACE amplification product, whereas the 5′ terminus sequence was determined after cloning of the 5′ RACE product in *E. coli* using the pGEM-T Easy Vector system. The complete PcRV genome was determined to be 13,604 nt long and was deposited in the GenBank database under accession number OP903920. The complete 13,604 nt genome of PcRV had a 156-nt untranslated region (UTR) at the 5′-terminus of the (+) strand and a 641-nt UTR at the 3′-end of the (+) strand; the first eight nucleotides at the 5′-end of the (-) strand genome, 5′-CAACCUAA-3′, were almost completely complementary to the last nine nucleotides of the (-) strand, leaving only the 3′-ultimate nucleotide of the PcRV genome, U, unpaired.

### 3.2. Phylogenetic Analysis

**Figure 3:**
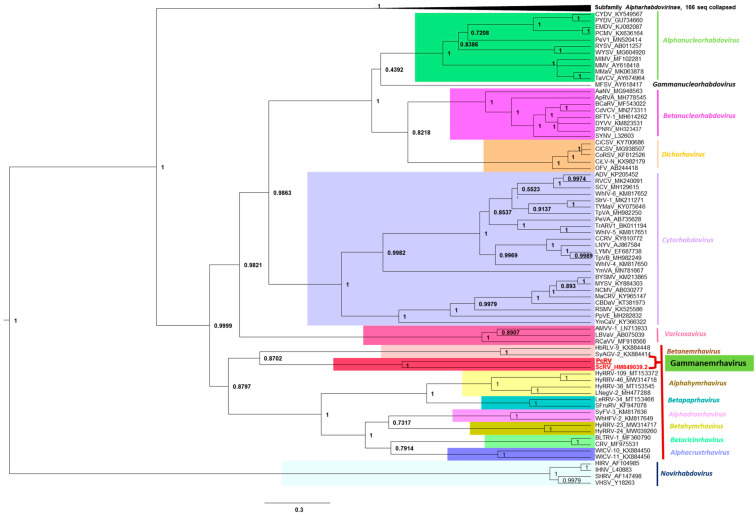
Phylogenetic analysis of full-length amino acid sequences of the rhabdovirus L proteins, with the addition of L-proteins of PcRV and ScRV. Maximum clade credibility (MCC) tree was inferred from MUSCLE alignments as described in Materials and Methods. Branch lengths are drawn to scale, with the scale bar showing the number of substitutions per site.

To clarify the phylogenetic position of the PcRV among all rhabdoviruses, the L-protein sequences of PcRV and ScRV were added to the alignment of L-proteins of all the approved members of the family *Rhabdoviridae* [25] and maximum clade credibility (MCC) tree was inferred from MUSCLE alignments (Figure 3). As can be seen from the phylogenetic tree topology (Figure 3), both PcRV and ScRV were placed close to each other in a separate lineage within the unassigned subfamily of rhabdoviruses, which currently comprises seven approved genera of rhabdoviruses [21,25]. This PcRV/ScRV lineage had a 100% support level and was placed as a sister clade with another lineage, genus *Betanemrhavirus*, comprising two nematode viruses, Hubei rhabdo-like virus 9 (HbRLV-9; KX884448) and Shayang ascaridia galli virus 2 (SyAGV-2; KX884414), both found in animal roundworms [26]. Given the high level of confidence in separation of the genus *Betanemrhavirus* from the PcRV/ScRV lineage (87%; see Figure 3), long branches of the two separate lineages in the phylogenetic tree, and a distinct genome organization between HbRLV-9/SyAGV-2 (six genes) and PcRV/ScRV (five genes), we propose to create a new genus comprising two viruses found in plant-parasitic cyst nematodes affecting potato (PcRV) and soybean (ScRV). Following the traditions in naming rhabdovirus taxa, we propose the name Gammanemrhavirus (see Figure 3).

### 3.3. PcRV Presence in Different Life Stages of G. Pallida, Prevalence over Time, and Testing for PCN Viruses in Other Globodera Species

**Figure 4:**
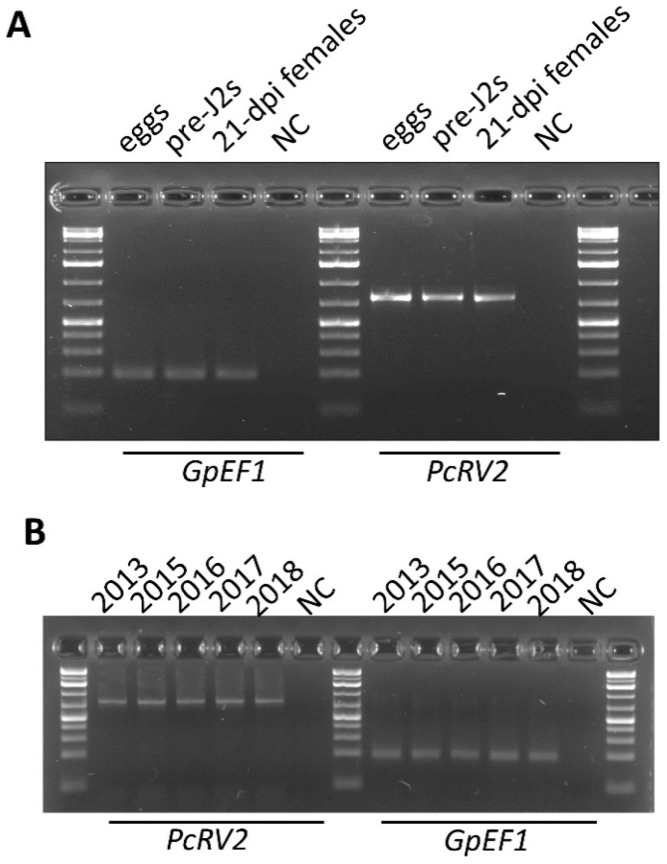
Detection of potato cyst nematode rhabdovirus (PcRV) across different life stages of *G. pallida* (**A**) and its prevalence in the inbred *G. pallida* populations (reared in 2013, 2015, 2016, 2017, and 2018) (**B**). *Globodera EF1* gene was used as a positive control. NC—negative non-template control. Pre-J2s—invasive juveniles. dpi—days post infection.

To check if PcRV was present in different developmental stages and different nematode populations, RT-PCR with PcRV2 primer set was used. Nematode EF1 gene amplification served as a positive control. PcRV-specific PCR products were obtained across all tested developmental stages, including *G. pallida* eggs, invasive J2s, and parasitic females at 21 days post infection (Figure 4A). Given the quarantine status of *G. pallida* in the US, the only available *G. pallida* samples to test were maintained at University of Idaho populations that originated from initial cysts collected in 2008 upon its single introduction in Idaho, USA. The RNA extracted from *G. pallida* populations reared over a period of 5 years, in 2013, 2015, 2016, 2017, and 2018, all showed RT-PCR amplification with PcRV2 primers (Figure 4B). Sanger sequencing of the amplified PCR products revealed no changes to the PcRV sequence over time.

**Figure 5:**
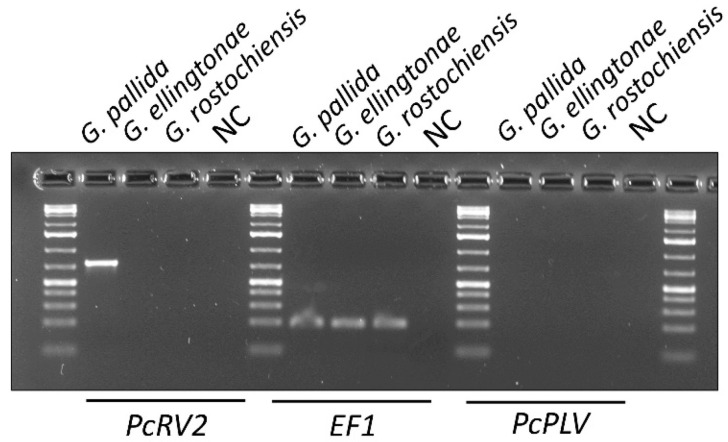
Detection of potato cyst nematode rhabdovirus (PcRV) and potato cyst nematode picorna-like virus (PcPLV, MG550273) in *Globodera* spp. nematodes present in the US and reproducing on potatoes. RT-PCR using RNA isolated from 10 cysts (*G. pallida*, *G. ellingtonae*, or *G. rostochiensis*) as a template and PcRV- or PcPLV-specific primers (see Supplementary Table S1). *Globodera EF1* gene was used as a positive control. NC—negative non-template control.

In addition to *G. pallida*, two other species of nematodes reproducing on potato, *G. rostochiensis* and *G. ellingtonae*, were tested for PcRV using RT-PCR with PcRV2 primers. PcRV presence was only detected in *G. pallida* samples, although strong amplification of nematode *EF1* gene, as a positive control, was observed across all the included *Globodera* spp. samples in the experiment (Figure 5). An attempt was made to test for a novel potato cyst nematode picorna-like virus (PcPLV) recently identified from the PCN transcriptome data of *Globodera* spp. [7]. RT-PCR-based screening for the presence of PcRLV in RNA extracted from *G. pallida*, *G. ellingtonae*, and *G. rostochiensis*, using PcPLV-specific primers as described previously [7], resulted in no RT-PCR bands amplified for all the tested samples, while the control EF-1-specific primers produced the expected 193-bp band (Supplementary Table S1; Figure 5).

## 4. Discussion

Plant-parasitic nematodes are heavy burdens on global food security. Use of microorganisms as biocontrol agents to manage plant parasitic nematodes is a promising approach, gaining more support over recent years. As obligate parasites, viruses can be viewed as a potential tool to control nematodes. For a long time, nematode viruses were neglected, primarily due to a lack of proper tools and approaches to address their presence. For example, in the late 1950s Loewenberg et al. (1959) first described the infection of root-knot nematodes with a virus-like pathogen supported by visible symptoms in nematodes, but no viral particles were visualized [27]. Although a number of electron microscopic studies reported virus-like particles in nematodes during the 1970s, due to a lack of biochemical or genomic analysis, these reports could not indisputably prove viral infections in nematodes [28,29,30]. It was not until the last decade that the advances in sequencing technologies and the reduction in costs for HTS have enabled and facilitated detection of naturally occurring viruses within free living nematodes, such as *C. elegans* and *C. briggsae* [31,32,33], as well as parasitic species of both animals [26,34] and plants (*Heterodera* spp., *Globodera* spp., and *Pratylenchus penetrans*) [7,22,35,36,37,38].

Rhabdoviruses are single-stranded, negative-sense RNA viruses with genomes ranging between 10 and 16 kb in size. Rhabdoviruses have enveloped, bullet-shaped, or bacilliform virion morphology, although unenveloped rhabdoviruses with filamentous virions have also been described [21]. In the latest update of the rhabdovirus taxonomy [21,25], the family *Rhabdoviridae* included 20 genera and 144 species found in vertebrate, arthropod, and plant hosts. Two genera of rhabdoviruses included species found in nematodes, *Alphanemrhavirus*, and *Betanemrhavirus*. Phylogenetically, alphanemrhaviruses and betanemrhaviruses were placed into two different sub-families, *Alpharhabdovirinae* and an unassigned sub-family, based on the sequences of their replication-associated L-proteins [25] (see also Figure 3). In 2011, a rhabdo-like virus genome was described assembled from the HTS data from soybean cyst nematode samples, named soybean cyst nematode rhabdovirus (ScRV; [22]). Phylogenetically, ScRV did not fit into any established genus of rhabdoviruses, including alphanemrhaviruses and betanemrhaviruses, and was left as an unassigned member of *Rhabdoviridae* [25]. Now, we demonstrated that a newly found PCN virus, PcRV, has very similar genome organization to ScRV (Figure 1) and is closely related to ScRV phylogenetically (Figure 3). Given the topology of the phylogenetic tree for the L-proteins, we proposed both ScRV and PcRV to form a new genus, tentatively named Gammanemrhavirus.

Detection of PcRV across different life stages, including eggs, preparasitic juveniles, and parasitic females, suggests vertical transmission as a possible route of infection. This observation is consistent with reports for other viruses of plant parasitic cyst nematodes [7,22,39] but not for root lesion nematode virus 1 (RLNV1) infecting the migratory species *Pratylenchus penetrans*, which was only detected in parasitic developmental stages, excluding eggs and juveniles [38]. Although vertical transmission has not been well studied for nematode viruses, this mode of transmission has been previously reported in insect viruses, such as the rhabdovirus Sigma virus infecting Drosophila [40]. Transovarial movement of the virus may be hypothesized to be an efficient means of transfer in cyst nematodes, whose life cycle is mostly sedentary except for the invasive J2 and adult male stages.

Given that our RNA samples were extracted from cultured *G. pallida* research populations reared over a period of 5 years, the PcRV infection seems to be long lived and stable in the nematode populations. It is worth noting that, although we attempted to extract RNA from limited cysts collected directly from the originally infested field in Idaho [4], the biological material was too degraded to obtain sufficient quality and quantity of nucleic acid for RT-PCR amplification. The *G. pallida* 2013 rearing population represents the first generation of nematodes bred in our greenhouse conditions, and it is reasonable to speculate that PcRV most likely has already been present in the original *G. pallida* field population.

Our RT-PCR testing confirmed presence of PcRV only in *G. pallida* but not in two other cyst nematode species capable of infecting potato, which are present in the US, *G. rostochiensis* and *G. ellingtonae*. In light of ScRV being detected in at least two nematode species from genera *Heterodera*, *H. glycines*, and *H. schachtii*, it was hypothesized that, due to a close phylogenetic relationship between these nematode species within one genus, viruses such as ScRV may have evolved within this subset of cyst nematodes before the species split from one another [39]. Thus, future studies examining the host status of *Globodera* spp. for PcRV are certainly warranted. Furthermore, picorna-like virus, PcPLV, identified previously in silico [7], was not detected with RT-PCR within our *Globodera* RNA samples. Notably, Ruark et al. [7] also failed in experimentally confirming the presence of this virus in *G. rostochiensis* RNA samples available to them. Interestingly, the HTS data used to identify the PcPLV genome were obtained from the Scottish population of *G. pallida*, where the PcRV genome was also discovered by us. Apparently, two identical or almost identical isolates of PcRV, PcRV-Id and PcRV-Sc, are circulating in the laboratory populations of *G. pallida* maintained in two geographically distant locations, Idaho and Scotland. Incidentally, the latter population of *G. pallida* maintained in Scotland is also infected with another virus, PcPLV [7], which is not present in the Idaho populations of PCN (Figure 5). This shows that viral infections of nematodes vary by geographic region and population. Alternatively, it is also possible that viral PcPLV titers in the Idaho populations are below detectible limits and the samples falsely appear virus-free.

Viral infections of plant parasitic nematodes prove to be a challenging research topic due to the ecological complexity within an agricultural system. Nematode virology represents a relatively new field, and this study further advances knowledge in this area. The effects of viruses on fitness of nematodes, viral tropism, and mechanisms driving viral transmission are largely unknown. Future research aiming at better understanding the effects of viruses infecting nematodes will help to explore potential use of PcRV as a novel management strategy for potato cyst nematodes.

## 5. Conclusions

In this work, we reported the genome sequences of a new rhabdovirus, PcRV, found in *G. pallida* populations maintained under quarantine in Idaho. The presence of this virus was validated and confirmed for the different life stages of *G. pallida*, indicating a vertical transmission mode. The overall organization of the 13,604 nt genome of PcRV and phylogenetic analysis of the RdRP suggest a close relationship between PcRV and another rhabdovirus, ScRV, from soybean cyst nematode. To the best of our knowledge, this is the first experimental evidence of a virus found in a potato cyst nematode. Another isolate of PcRV was identified among the publicly available transcriptomics outputs generated from the Scottish populations of *G. pallida*, with a genome sequence almost identical to the Idaho isolate of PcRV. Based on the phylogenetic position of the PcRV, we propose to create a new genus with a tentative name Gammanemrhavirus, comprising two viruses found in plant cyst nematodes, PcRV and ScRV.

## Figures and Tables

**Table 1:** Pairwise comparison of protein sequences for potato cyst nematode rhabdovirus (PcRV) and soybean cyst nematode virus (ScRV).

Gene/Protein	Protein Size in PcRV, aa	Amino Acid Identity, %	Coverage, %
ORF 1/N	593	21.2	46.0
ORF 2/P	329	21.4	54.0
ORF 3/? ^(1)^	180	- ^(2)^	-
ORF 4/G	549	21.3	71.0
ORF 5/L	2180	34.7	84.0

^(1)^ ? = protein with an unknown function; ^(2)^ - = no significant similarity.

## Data Availability

PcRV whole genome sequences determined in this study were deposited in the GenBank database under accession numbers OP903920 and BK062887.

## Supplementary Materials

The following are available online at https://www.mdpi.com/article/10.3390/v14122718/s1, Table S1: Potato cyst nematode rhabdovirus (PcRV)-specific primers used for RT-PCR amplifications and sequencing of the PcRV genome.
